# Angiogenesis is an independent prognostic factor in malignant mesothelioma

**DOI:** 10.1054/bjoc.2001.1997

**Published:** 2001-09

**Authors:** J G Edwards, G Cox, A Andi, J L Jones, R A Walker, D A Waller, K J O'Byrne

**Affiliations:** University Department of Medical Oncology, Leicester Royal Infirmary, Osborne Building, Leicester, LE1 5WW, UK; Department of Respiratory Medicine and Thoracic Surgery, Glenfield Hospital, Groby Road, Leicester, LE3 9QP, UK; Department of Pathology, University of Leicester, Leicester Royal Infirmary, Leicester, LE1 5WW, UK

**Keywords:** malignant mesothelioma, angiogenesis, prognosis, staging

## Abstract

Angiogenesis is essential for tumour growth beyond 1 to 2 mm in diameter. The clinical relevance of angiogenesis, as assessed by microvessel density (MVD), is unclear in malignant mesothelioma (MM). Immunohistochemistry was performed on 104 archival, paraffin-embedded, surgically resected MM samples with an anti-CD34 monoclonal antibody, using the Streptavidin–biotin complex immunoperoxidase technique. 93 cases were suitable for microvessel quantification. MVD was obtained from 3 intratumoural hotspots, using a Chalkley eyepiece graticule at × 250 power. MVD was correlated with survival by Kaplan–Meier and log-rank analysis. A stepwise, multivariate Cox model was used to compare MVD with known prognostic factors and the EORTC and CALGB prognostic scoring systems. Overall median survival from the date of diagnosis was 5.0 months. Increasing MVD was a poor prognostic factor in univariate analysis (*P* = 0.02). Independent indicators of poor prognosis in multivariate analysis were non-epithelial cell type (*P* = 0.002), performance status > 0 (*P* = 0.003) and increasing MVD (*P* = 0.01). In multivariate Cox analysis, MVD contributed independently to the EORTC (*P* = 0.006), but not to the CALGB (*P* = 0.1), prognostic groups. Angiogenesis, as assessed by MVD, is a poor prognostic factor in MM, independent of other clinicopathological variables and the EORTC prognostic scoring system. Further work is required to assess the prognostic importance of angiogenic regulatory factors in this disease. http://www.bjcancer.com © 2001 Cancer Research Campaign  http://www.bjcancer.com


*British Journal of Cancer* (2001) **85**(6), 863–868
© 2001 Cancer Research Campaign
doi: 10.1054/ bjoc.2001.1997, available online at http://www.idealibrary.com on IDEAL
http://www.bjcancer.com

### Angiogenesis is an independent prognostic factor in malignant mesothelioma

**JG Edwards[1,2], G Cox[1,2], A Andi[1], JL Jones[3], RA Walker[3], DA Waller[2] and KJ O'Byrne[1]**

[1]University Department of Medical Oncology, Osborne Building, Leicester Royal Infirmary, Leicester LE1 5WW, UK; [2]Department of Respiratory Medicine and Thoracic Surgery, Glenfield Hospital, Groby Road, Leicester LE3 9QP, UK; [3]Department of Pathology, University of Leicester, Leicester Royal Infirmary, Leicester LE1 5WW, UK

**Summary** Angiogenesis is essential for tumour growth beyond 1 to 2 mm in diameter. The clinical relevance of angiogenesis, as assessed by microvessel density (MVD), is unclear in malignant mesothelioma (MM). Immunohistochemistry was performed on 104 archival, paraffin-embedded, surgically resected MM samples with an anti-CD34 monoclonal antibody, using the Streptavidin–biotin complex immunoperoxidase technique. 93 cases were suitable for microvessel quantification. MVD was obtained from 3 intratumoural hotspots, using a Chalkley eyepiece graticule at × 250 power. MVD was correlated with survival by Kaplan–Meier and log-rank analysis. A stepwise, multivariate Cox model was used to compare MVD with known prognostic factors and the EORTC and CALGB prognostic scoring systems. Overall median survival from the date of diagnosis was 5.0 months. Increasing MVD was a poor prognostic factor in univariate analysis ($P = 0.02$). Independent indicators of poor prognosis in multivariate analysis were non-epithelial cell type ($P = 0.002$), performance status > 0 ($P = 0.003$) and increasing MVD ($P = 0.01$). In multivariate Cox analysis, MVD contributed independently to the EORTC ($P = 0.006$), but not to the CALGB ($P = 0.1$), prognostic groups. Angiogenesis, as assessed by MVD, is a poor prognostic factor in MM, independent of other clinicopathological variables and the EORTC prognostic scoring system. Further work is required to assess the prognostic importance of angiogenic regulatory factors in this disease. © 2001 Cancer Research Campaign http://www.bjcancer.com

**Keywords**: malignant mesothelioma; angiogenesis; prognosis; staging

Malignant mesothelioma (MM) is a fatal cancer of increasing incidence associated with asbestos exposure (Peto et al, 1999). MM responds poorly to aggressive conventional therapy (Sterman et al, 1999) and has an appalling prognosis. Median survival in the United Kingdom, where management has been typically palliative, is between 6 and 12 months from the time of onset of symptoms (Law et al, 1984; McLean and Patel, 1997; Edwards et al, 2000). Pathological tumour, nodes and metastasis (TNM) staging is difficult to achieve. In a large series of patients undergoing extrapleural pneumonectomy and adjuvant chemoradiotherapy, International Mesothelioma Interest Group (IMIG) TNM staging (Rusch, 1995) failed to stratify survival (Sugarbaker et al, 1999), questioning the value of this approach in predicting outcome. Biological markers of prognosis have attracted interest in other solid tumours and may provide prognostic information independent from TNM stage (Cox et al, 2000a, 2001).

Angiogenesis is the formation of new blood vessels from existing vasculature, during which normally quiescent endothelial cells proliferate and gain invasive characteristics. Angiogenesis is necessary for tumour growth of greater than 1 to 2 mm in diameter (Hanahan and Folkman, 1996). High intratumoural microvessel counts, and indirect measure of the intensity of angiogenesis, are associated with a poor prognosis in solid tumours (Fox et al, 1995; Giatromanolaki et al, 1996; Cox et al, 2000b). There have been preliminary reports of the prognostic value of microvessel counts in malignant mesothelioma (Kumar-Singh et al, 1997; Ohta et al, 1999). These relatively small studies, of 25 and 54 cases, have suggested a relationship between increased microvessel counts and poor prognosis. However methodology, the mean vessel count obtained and statistical significance varied greatly between the studies.

This study incorporated the conclusions of a consensus paper on the evaluation of tumour angiogenesis into our methodology (Vermeulen et al, 1996) and evaluated microvessel density (MVD) in 104 cases of MM. The prognostic significance of MVD was examined in a multivariate model, incorporating clinical and pathological factors. The contribution of MVD to the Cancer and Leukemia Group B (CALGB) (Herndon et al, 1998) and European Organisation for the Research and Treatment of Cancer (EORTC) (Curran et al, 1998) prognostic scoring systems, which we have validated previously in this cohort of patients (Edwards et al, 2000), was analysed.

### MATERIALS AND METHODS

### Patients

All cases of MM presenting to our institution since 1988 were identified and case notes reviewed. Relevant demographic, clinical and pathological data were retrieved. Clinicopathological prognostic factors, including CALGB (Herndon et al, 1998) and EORTC (Curran et al, 1998) prognostic groups were assessed, as described previously (Edwards et al, 2000). The majority of patients were referred to the regional Department of

*Received 1 March 2001*
*Revised 30 May 2001*
*Accepted 12 June 2001*

*Correspondence to:* KJ O'Byrne

Cardiothoracic Surgery for surgical biopsy, management of pleural effusion or empyema, or for radical surgery. The detailed histopathological report was obtained for each case and the slides reviewed by a pathologist to both confirm the diagnosis and to assess the most suitable block for microvessel quantification. One block was selected and a single histological section stained, as this has been shown to be representative of tumour angiogenesis as a whole in breast cancer (Martin et al, 1997b). Cancer-specific survival was calculated from the date of the diagnostic biopsy. Pre-diagnostic variables, such as performance status and haematological indices, were taken from immediately before this time.

### Immunohistochemistry

Representative formalin-fixed paraffin-embedded blocks of tumour were chosen for each case. 4 μm sections were cut onto glass slides previously treated with 2% 3-aminopropylethoxysilane. Sections were dewaxed in xylene and rehydrated through graded alcohols. Endogenous peroxidase activity was blocked by immersion in 2% hydrogen peroxide for 30 minutes. Sections were rinsed in deionised water followed by tris-buffered saline (TBS) containing 0.1% bovine serum albumin. Non-specific staining was blocked by incubation with 20% normal rabbit serum for 10 minutes. Sections were incubated overnight at 4˚C with CD34 antibody (NCL-END, Novocastra, Newcastle, UK) at a dilution of 1 in 50. Following washing in TBS, sections were incubated for 30 minutes with a biotinylated rabbit anti-mouse whole immunoglobulin secondary antibody (E0354, Dako, Ely, UK). Sections were rinsed in TBS before incubation with streptavidin–biotin peroxidase complex (K0377, Dako) for 30 minutes. Finally, sections were rinsed in TBS and incubated with the chromogen diaminobenzidine tetrahydrochloride for 10 minutes before counterstaining with haematoxylin. Sections were dehydrated through graded alcohols and mounted in resinous mountant. Negative controls had the primary antibody omitted, whilst microvessels from surrounding normal lung were used as an internal positive control.

### Microvessel quantification

Angiogenesis was assessed indirectly with the aid of a Chalkley eyepiece graticule, as previously described (Cox et al, 2000b). Each section was examined under low power to identify 3 intratumoural microvessel ‘hot spots’. These areas were then examined at × 250 magnification using a 25-point Chalkley eyepiece graticule. The Chalkley graticule was orientated so that the maximum number of points coincided with immunostained structures. Structures with the morphological features of microvessels that stained with the chromogen, irrespective of whether a lumen was present, were counted. MVD was defined in this study as the sum of the number of points thus counted from 3 hot spots. The Chalkley graticule covers an area of 0.115 $mm^2$ at × 250 magnification. Sections were analysed by 2 investigators blinded to clinicopathological factors and outcome.

### Statistical analysis

Statistical analysis was performed using the SPSS software system (SPSS for Windows Version 9.0, SPSS Inc, Chicago, USA). Differences in total Chalkley count within categorical prognostic factors were assessed with Student’s *t*-test. Linear regression analysis was used to assess correlations with continuous prognostic variables. Cancer-specific survival curves were estimated using the Kaplan–Meier method and the log-rank test was used to assess the statistical significance of differences between groups. A Cox proportional hazards regression model was used to identify statistically significant differences in survival and estimate hazard ratios and 95% confidence intervals (CI) (Cox, 1972). The assumption of proportional hazards was assessed graphically by plotting log[-log(survivor)] against log(time) for each of the prognostic groups. Prognostic variables identified by univariate analysis, with $P < 0.1$, were analysed in a multivariate Cox model. Cases in which complete prognostic data retrieval was not possible (due to missing or destroyed case notes, or missing data within case notes that had been inspected) were excluded from multivariate analysis. A forward, stepwise selection procedure was used, with variables being added to the model according to a partial likelihood ratio test, using an entry criterion of $P < 0.05$.

### RESULTS

### Patient characteristics and immunohistochemistry results

In all cases, microvessels stained for CD34 but tumour cells did not. Figures 1 and 2 show examples of anti-CD34 immunostaining with a high and low MVD. Of the 140 cases of MM presenting to our department, 36 had insufficient material for microvessel counting (i.e. less than 3 full high power fields of tumour). In the remaining 104 cases, immunostaining of stromal elements, which had the morphological features of myofibroblasts, was present in 18 (17%) cases (Figure 3). Microvessel quantification was only carried out in 7 of these cases in which the morphological pattern of stromal staining was clearly distinct from that of microvessels. Stromal staining therefore precluded microvessel quantification in 11 (11%) cases. In the 93 cases successfully assessed with microvessel counting the surgical procedures performed were biopsy alone (54 cases), parietal pleurectomy (18), decortication (33) and extrapleural pneumonectomy (8). Immunohistochemistry was required in 52% of these cases to confirm the diagnosis. The most commonly used markers were CEA, BerEP4, AUA-1, cytokeratin, thrombomodulin, HBME-1 and CAM 5.2 in 41, 38, 27, 22, 19 and 16 cases respectively. Of the 93 cases in which MVD was derived, cell type was epithelioid in 48, mixed cellularity in 18 and sarcomatoid in 27. It did not prove possible to derive an accurate IMIG TNM stage from this cohort of patients: 58% of patients underwent computed tomography, but pathological verification of stage was only available in 20 (21.5%) patients.

### Microvessel quantification

The median Chalkley count (sum of 3 hotspots) in the 93 cases was 23 (range 13–29). There was no significant difference in Chalkley count between epithelial and non-epithelial cell types, nor within other categorical prognostic factors (data not shown). However, a trend towards a positive correlation between cases with vessel counts ≥ median and weight loss of greater than 5% ($P = 0.055$) was noted. With linear regression analysis, a significant correlation was seen between increasing MVD and platelet count ($P = 0.048$, Table 1), but not with white cell count, haemoglobin or age. Furthermore a positive correlation was seen between high platelet counts and weight loss > 5% ($P < 0.0001$). There was

 © 2001 Cancer Research Campaign

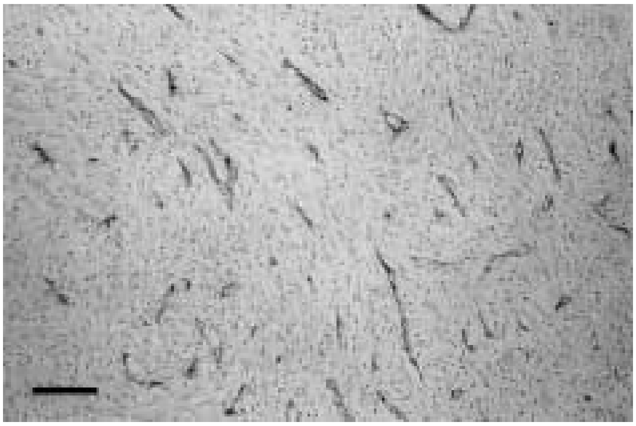

**Figure 1** Photomicrograph of a tumour section with a high microvessel density (× 200, bar = 50 µm)

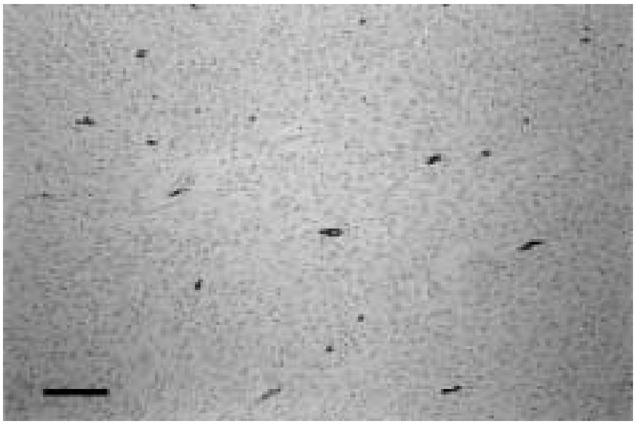

**Figure 2** Photomicrograph of a tumour section with a low microvessel density (× 200, bar = 50 µm)

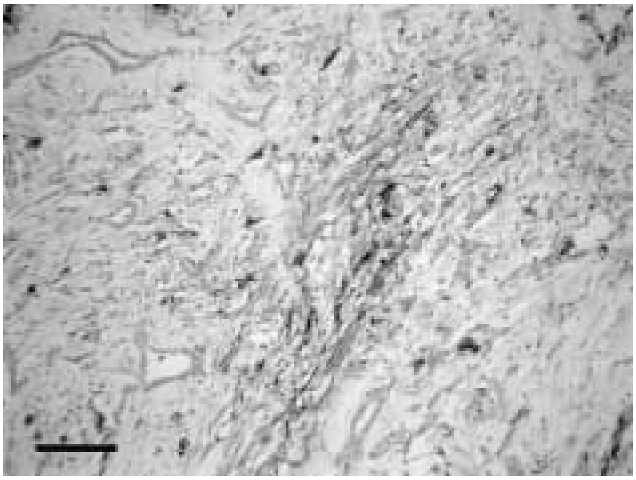

**Figure 3** Photomicrograph of a tumour section displaying stromal anti-CD34 immunostaining, which prevented accurate assessment of microvessel density (× 250, bar = 50 µm)

no significant difference in MVD between the high and low-risk groups of the EORTC prognostic scoring system. With regard to the CALGB system, even numbered groups displayed small numbers and so groups were combined for statistical analysis. Although a significant difference in MVD was noted between Groups 3 and 4 compared to Groups 5 and 6, there was no significant overall trend towards increased MVD in the higher risk groups.

**Table 1** Correlation of clinicopathological prognostic factors with MVD by linear regression analysis

| | *r* | *P* |
|---|---|---|
| WBC | 0.06 | 0.57 |
| Platelets | 0.21 | 0.048 |
| Haemoglobin | 0.04 | 0.71 |
| Age | 0.06 | 0.53 |

### Survival

Overall median survival from the date of histological diagnosis for the 93 cases was 5.0 months. 12 cases died within 30 days. Excluding these cases did not have a significant effect on any of the survival analyses: therefore all 93 cases were included. When entered into a Cox proportional hazards model, high MVD was a poor prognostic factor as a continuous variable ($P = 0.02$) and as a categorical variable at cut points from the 20th centile up to the median ($P = 0.007$) (Figure 4). Other statistically significant poor prognostic factors by univariate Cox proportional hazards analysis were increasing age ($P = 0.03$), weight loss > 5% ($P = 0.002$), presence of pleuritic chest pain ($P = 0.03$), Eastern Co-operative Oncology Group (ECOG) performance status > 0 ($P < 0.0001$), white blood count (WBC) > $8.3 \times 10^9$ $l^{-1}$ ($P = 0.02$) and cell type ($P = 0.0001$, Table 2). 10 cases were excluded from multivariate analysis due to missing prognostic data in case notes. In multivariate analysis, non-epithelial cell type was the strongest independent risk factor followed by performance status > 0 and increasing MVD (Table 3). When tested against the prognostic scoring systems in Cox multivariate analysis, increasing MVD contributed independently to the EORTC system ($P = 0.006$) but not the CALGB system ($P = 0.1$, Table 4).

### DISCUSSION

This study demonstrates that increased MVD, as assessed by Chalkley counting, is an independent prognostic factor in MM. This is in agreement with other solid tumours and with 2 previous reports in MM (Kumar-Singh et al, 1997; Ohta et al, 1999). Kumar Singh et al found that MVD was a significant prognostic factor in univariate analysis and was independent of MM cell type, tumour grade and patient age in multivariate analysis (Kumar-Singh et al, 1997). However, total microvessel area, when calculated from computer-aided image analysis, was not a significant prognostic factor, thus suggesting that the size of microvessels is not as important as their number. Ohta et al examined both MVD and lymphatic vessel density in 54 tumours and found statistically insignificant trends between high MVD and both poor survival and positive lymph node status. In multivariate analysis, gender, IMIG stage and high MVD were significant independent poor prognostic factors. In a further study, Tolnay et al noted that MVD correlated with expression of the angiogenic growth factor hepatocyte growth factor/scatter factor (HGF/SF) but did not comment on any relationship to prognosis (Tolnay et al, 1998).

The protocol for this study was based on the findings of an international consenus paper on the quantification of angiogenesis in solid tumours (Vermeulen et al, 1996). This suggested that manual

© 2001 Cancer Research Campaign

**Table 2** Prognostic factors analysed in a univariate Cox proportional hazards model, for cases with satisfactory MVD assessment ($n$ = 93)

| Variable | | *n* | Hazard ratio | Hazard ratio 95% confidence intervals | | *P* |
|---|---|---|---|---|---|---|
| Gender | Female | 8 | | | | |
| | Male | 85 | 2.19 | 0.95 | 5.07 | 0.07 |
| Age | | 93 | 1.03 | 1.00 | 1.05 | 0.03 |
| Weight loss | No | 38 | | | | |
| | Yes | 47 | 2.02 | 1.28 | 3.19 | 0.002 |
| Asbestos exposure | No | 14 | | | | |
| | Yes | 61 | 0.70 | 0.38 | 1.26 | 0.2 |
| Chest pain | No | 22 | | | | |
| | Yes | 66 | 1.79 | 1.07 | 3.00 | 0.03 |
| ECOG performance status | 0 | 37 | | | | |
| | 1 or 2 | 52 | 2.61 | 1.66 | 4.11 | < 0.001 |
| WBC | $< 8.3 \times 10^9$ l$^{-1}$ | 25 | | | | |
| | $> 8.3 \times 10^9$ l$^{-1}$ | 61 | 1.77 | 1.08 | 2.90 | 0.02 |
| Platelets | $< 400 \times 10^9$ l$^{-1}$ | 51 | | | | |
| | $> 400 \times 10^9$ l$^{-1}$ | 35 | 1.48 | 0.95 | 2.31 | 0.09 |
| Haemoglobin | > 14 g dl$^{-1}$ | 56 | | | | |
| | < 14 g dl$^{-1}$ | 31 | 1.39 | 0.87 | 2.22 | 0.2 |
| Cell Type | Epithelial | 47 | | | | |
| | Mixed or sarcomatoid | 46 | 2.46 | 1.57 | 3.85 | 0.0001 |
| Surgical resection | Yes | 55 | | | | |
| | No | 38 | 1.33 | 0.82 | 2.16 | 0.2 |
| EORTC | Low risk | 31 | | | | |
| | High risk | 56 | 2.33 | 1.45 | 3.74 | 0.0005 |
| CALGB | Groups 1 and 2 | 14 | | | | |
| | Groups 3 and 4 | 41 | 2.39 | 1.14 | 5.00 | 0.02 |
| | Groups 5 and 6 | 31 | 6.51 | 2.94 | 14.39 | < 0.0001 |
| Microvessel density | | 93 | 1.03 | 1.01 | 1.05 | 0.02 |

**Table 3** Significant prognostic variables identified in a forward, stepwise, multivariate Cox proportional hazards model (MVD was analysed as a continuous variable)

| Factor | Hazard ratio | 95% Confidence interval | *P* |
|---|---|---|---|
| Non-epithelial cell type | 2.23 | 1.35–3.67 | 0.002 |
| Performance status > 0 | 2.10 | 1.29–3.43 | 0.003 |
| MVD | 1.04 | 1.01–1.06 | 0.01 |

**Table 4** Contribution of MVD to CALGB and EORTC prognostic scoring systems in multivariate Cox proportional hazards analysis

| Factor | | Hazard ratio | 95% Confidence interval | *P* |
|---|---|---|---|---|
| CALGB | Groups 1/2 | 1 | | |
| | Groups 3/4 | 2.39 | 1.14–5.00 | |
| | Groups 5/6 | 6.50 | 2.94–14.39 | < 0.0001 |
| MVD | | | | 0.1 |
| EORTC | Low-risk group | 1 | | |
| | High-risk group | 2.42 | 1.50–3.90 | 0.0003 |
| MVD | 1.04 | 1.01–1.06 | 0.006 | |

vessel counting in hot spots was the appropriate method for assessing angiogenesis objectively in tumours and that either anti-CD34 or CD31 antibodies should be used. The anti-CD34 monoclonal antibody was chosen for this study as it has been shown to give more reproducible immunostaining of microvessels than either the anti-CD31 or anti-Factor VIII monoclonal antibodies in breast cancer (Martin et al, 1997a). This choice was supported by Kumar-Singh et al who found that CD34 staining was better delineated and easier to assess than CD31 in MM (Kumar-Singh et al, 1997). Stromal staining was seen in 18 cases in our series. In 7 cases the pattern of stromal staining was clearly morphologically distinct from that seen in microvessels and these were included in the analysis. Kumar-Singh described a ‘perivascular wash’ in some cases with anti-CD34 immunohistochemistry, but did not report staining of specific stromal elements. In no case was MM tumour-cell CD 34 positivity seen, in keeping with a recent report (Attanoos et al, 2000). The Chalkley counting method of MVD assessment was chosen because it has been shown to be a rapid and objective method of MVD assessment in breast (Fox et al, 1995; Hansen et al, 2000), bladder (Dickinson et al, 1994; Chaudhary et al, 1999) and non-small-cell lung cancers (Giatromanolaki et al, 1996; Cox et al, 2000b).

A significant correlation with the platelet count was seen in this study. This supports the hypothesis that platelets may play an important role in tumour angiogenesis. Platelets are an important source of angiogenic growth factors such as vascular endothelial growth factor (VEGF) and basic fibroblast growth factor (bFGF) (Banks et al, 1998; Pinedo et al, 1998). Platelets are likely to adhere to intratumoural endothelium, which can lead to platelet activation and the release and accumulation of high local concentrations of these growth factors (Pinedo et al, 1998; Verheul et al, 2000). A positive correlation has been established between serum VEGF and platelet count in cancer patients (Vermeulen et al,

 *© 2001 Cancer Research Campaign*

1999) and these have been further correlated to prognosis in solid tumours (O'Byrne et al, 1999).

Accurate TNM staging is difficult to achieve in the majority of patients with MM. Only a small proportion of patients are suitable for radical surgery, which does allow accurate pathological TNM staging, but even the validity of the International Mesothelioma Interest Group TNM staging system has been questioned (Sugarbaker et al, 1999). Therefore, biological markers may have an important role in providing prognostic information, which is not only of use in individual cases, but also crucial to the design and interpretation of clinical trials. This study clearly demonstrates that MVD is an independent prognostic factor in MM, which also contributes significantly to the EORTC prognostic scoring system. It is possible that the lack of contribution to the CALGB prognostic groups is due to the inclusion of weight loss as a parameter in this prognostic system. We found a near significant correlation between high MVD and weight loss. Weight loss in cancer patients is associated with raised inflammatory cytokine levels, including interleukin (IL)-6 (Scott et al, 1996). IL-6 is an angiogenic growth factor (Motro et al, 1990). Serum IL-6 levels correlate with the platelet count in MM (Nakano et al, 1998). These observations are in keeping with the finding of a positive correlation between platelet count and both MVD and weight loss in our patient series.

Other biological prognostic markers in MM include the cytokeratin marker Cyfra 21–1 (Schouwink et al, 1999), syndecan-1 (Kumar-Singh et al, 1998), bFGF (Kumar-Singh et al, 1999) and Simian virus-40 sequences (Procopio et al, 2000). We have recently presented data in operable non-small-cell lung cancer indicating that MVD contributes to a biological prognostic model which is independent of TNM stage (Cox et al, 2001). Using a similar approach, it may be possible to create a biological staging system in MM, which would avoid the current difficulties in predicting outcome associated with TNM staging. In addition to bFGF and IL-6, a number of other angiogenic factors have been studied in MM, although correlation between their expression and angiogenesis is poorly understood. These include HGF/SF (Tolnay et al, 1998), IL-8 (Antony et al, 1996) and urokinase plasminogen activator (Shetty et al, 1995). VEGF expression was correlated to MVD in MM by Ohta (Ohta et al, 1999) but not in the Kumar-Singh study (Kumar-Singh et al, 1999). However VEGF expression was not found to be a significant poor prognostic factor in either study.

Research into the mechanisms underlying angiogenesis has resulted in the discovery of a number of potential anti-angiogenic agents and endogenous angiostatic peptides, which are currently undergoing investigation in solid tumours (Bicknell and Harris, 1996; Twardowski and Gradishar, 1997; Cherrington et al, 2000; Eatock et al, 2000; O'Byrne et al, 2000; Talks and Harris, 2000). These include: synthetic matrix metalloproteinase inhibitors (e.g. Batimastat (Macaulay et al, 1999) and Marimastat (Steward, 1999)), cytokines and their modulators (e.g. interferon α-2a, IL-12 (Duda et al, 2000) and thalidomide (Calabrese and Fleischer, 2000)) and angiostatic factors (e.g. angiostatin (O'Reilly et al, 1994), endostatin (O'Reilly et al, 1997), TNP-470 (Gervaz and Fontolliet, 1998) and platelet factor-4 (Gupta et al, 1995)).

In conclusion, assessment of angiogenesis may have an important role in the prognostic evaluation of MM and contribute to currently established prognostic scoring systems. Investigation of the mechanisms of angiogenesis in MM may provide further prognostic information and help to rationalise therapy. Such markers may be useful in the selection of patients for radical surgery and chemotherapeutic treatment protocols including the use of anti-angiogenic agents.

### ACKNOWLEDGEMENTS

JGE is supported by a Leicester Royal Infirmary Research Fellowship. We acknowledge the support of the Institute of Cancer Studies and the Institute for Lung Health, Leicester. This study was funded by a Glenfield Hospital Research and Development Grant, the June Hancock Memorial Fund and the Sir Samuel Scott of Yews Trust. We thank Dr KR Abrams, Department of Epidemiology & Public Health, University of Leicester, for his statistical advice.

### REFERENCES

Antony VB, Hott JW, Godbey SW and Holm K (1996) Angiogenesis in mesotheliomas. Role of mesothelial cell derived IL-8. *Chest* **109**: 21S–22S

Attanoos RL, Suvarna SK, Rhead E, Stephens M, Locke TJ, Sheppard MN, Pooley FD and Gibbs AR (2000) Malignant vascular tumours of the pleura in "asbestos" workers and endothelial differentiation in malignant mesothelioma. *Thorax* **55**: 860–863

Banks R, Forbes M, Kinsey S, Stanley A, Ingham E, Walters C and Selby P (1998) Release of angiogenic cytokine vascular endothelial growth factor (VEGF) from platelets: significance for VEGF measurements and cancer biology. *Brit J Cancer* **77**: 956–964

Bicknell R and Harris AL (1996) Mechanisms and therapeutic implications of angiogenesis. *Current Opinions in Oncology* **8**: 60–65

Calabrese L and Fleischer AB (2000) Thalidomide: current and potential clinical applications. *Am J Med* **108**: 487–495

Chaudhary R, Bromley M, Clarke NW, Betts CD, Barnard RJ, Ryder WD and Kumar S (1999) Prognostic relevance of micro-vessel density in cancer of the urinary bladder. *Anticancer Res* **19**: 3479–3484

Cherrington JM, Strawn LM and Shawver LK (2000) New paradigms for the treatment of cancer: the role of anti-angiogenesis agents. *Adv Cancer Res* **79**: 1–38

Cox D (1972) Regression models and life tables. *Journal of the Royal Statistical Society (B)* **34**: 187–220

Cox G, Jones JL, Andi A, Waller DA and O' Byrne KJ (2001) A biological staging model for operable non-small cell lung cancer. *Thorax* **56**: 561–566

Cox G, Jones JL and O'Byrne KJ (2000a) Matrix metalloproteinase 9 and the epidermal growth factor signal pathway in operable non-small cell lung cancer. *Clin Cancer Res* **6**: 2349–2355

Cox G, Walker RA, Andi A, Steward WP and O'Byrne KJ (2000b) Prognostic significance of platelet and microvessel counts in operable non-small cell lung cancer. *Lung Cancer* **29**: 169–177

Curran D, Sahmoud T, Therasse P, Van Meerbeeck J, Postmus PE and Giaccone G (1998) Prognostic factors in patients with pleural mesothelioma: The European Organisation for Research and Treatment of Cancer Experience. *Journal of Clinical Oncology* **16**: 145–152

Dickinson AJ, Fox SB, Persad RA, Hollyer J, Sibley GN and Harris AL (1994) Quantification of angiogenesis as an independent predictor of prognosis in invasive bladder carcinomas. *Br J Urol* **74**: 762–766

Duda DG, Sunamura M, Lozonschi L, Kodama T, Egawa S, Matsumoto G, Shimamura H, Shibuya K, Takeda K and Matsuno S (2000) Direct in vitro evidence and in vivo analysis of the antiangiogenesis effects of interleukin 12. *Cancer Res* **60**: 1111–1116

Eatock MM, Schatzlein A and Kaye SB (2000) Tumour vasculature as a target for anticancer therapy. *Cancer Treat Rev* **26**: 191–204

Edwards JG, Abrams KR, Leverment JN, Spyt TJ, Waller DA and O'Byrne KJ (2000) Prognostic factors for malignant mesothelioma in 142 patients: validation of CALGB and EORTC prognostic scoring systems. *Thorax* **55**: 731–735

Fox SB, Leek RD, Weekes MP, Whitehouse RM, Gatter KC and Harris AL (1995) Quantitation and prognostic value of breast cancer angiogenesis: comparison of microvessel density, Chalkley count, and computer image analysis. *Journal of Pathology* **177**: 275–283

Gervaz P and Fontolliet C (1998) Therapeutic potential of the anti-angiogenesis drug TNP-470. *Int J Exp Pathol* **79**: 359–362

Giatromanolaki A, Koukourakis M, O'Byrne K, Fox S, Whitehouse R, Talbot DC, Harris AL and Gatter KC (1996) Prognostic value of angiogenesis in operable non-small cell lung cancer. *J Pathol* **179**: 80–88

© 2001 Cancer Research Campaign 

Gupta SK, Hassel T and Pal Singh J (1995) A potent inhibitor of endothelial cell proliferation is generated by proteolytic cleavage of the chemokine platelet factor 4. *Proc Nat Acad Sci USA* **92**: 7799–7803

Hanahan D and Folkman J (1996) Patterns and emerging mechanisms of the angiogenic switch during tumorigenesis. *Cell* **86**: 353–364

Hansen S, Grabau DA, Sorensen FB, Bak M, Vach W and Rose C (2000) The prognostic value of angiogenesis by Chalkley counting in a confirmatory study design on 836 breast cancer patients. *Clin Cancer Res* **6**: 139–146

Herndon JE, Green MR, Chahinian AP, Corson JM, Suzuki Y and Vogelzang NJ (1998) Factors predictive of survival among 337 patients with mesothelioma treated between 1984 and 1994 by the Cancer and Leukemia Group B. *Chest* **113**: 723–731

Kumar-Singh S, Vermeulen PB, Weyler J, Segers K, Weyn B, Van Daele A, Dirix LY, Van Oosterom AT and Van Marck E (1997) Evaluation of tumour angiogenesis as a prognostic marker in malignant mesothelioma. *J Pathol* **182**: 211–216

Kumar-Singh S, Jacobs W, Dhaene K, Weyn B, Bogers J, Weyler J and Van Marck E (1998) Syndecan-1 expression in malignant mesothelioma: correlation with cell differentiation, WT1 expression, and clinical outcome. *J Pathol* **186**: 300–305

Kumar-Singh S, Weyler J, Martin KJ, Vermeulen PB and Van Marck E (1999) Angiogenic cytokines in mesothelioma: a study of VEGF, FGF-1 and -2, and TGF beta expression. *J Pathol* **189**: 72–78

Law M, Gregor A, Hodson ME, Bloom H and Turner-Warwick M (1984) Malignant mesothelioma of the pleura: a study of 52 treated and 64 untreated patients. *Thorax* **39**: 255–259

Macaulay V, O'Byrne K, Saunders M, Braybrooke J, Long L, Gleeson F, Mason C, Harris A, Brown P and Talbot D (1999) Phase I study of intrapleural batimastat (BB-94), a matrix metalloproteinase inhibitor, in the treatment of malignant pleural effusions. *Clin Cancer Res* **5**: 513–520

Martin L, Green B, Renshaw C, Lowe D, Rudland P, Leinster S and Winstanley J (1997a) Examining the technique of angiogenesis assessment in invasive breast cancer. *Brit J Cancer* **76**: 1046–1054

Martin L, Holcombe C, Green B, Leinster S and Winstanley J (1997b) Is a histological section representative of whole tumour vascularity in breast cancer? *Brit J Cancer* **76**: 40–43

McLean AN and Patel KR (1997) Clinical features and epidemiology of malignant pleural mesothelioma in west Glasgow 1987–1992. *Scot Med J* **42**: 37–39

Motro B, Itin A, Sachs L and Keshet E (1990) Pattern of Interleukin 6 gene expression in vivo suggests a role for this cytokine in angiogenesis. *Proc Natl Acad Sci USA* **87**: 3092–3096

Nakano T, Chahinian A, Shinjo M, Tonomura A, Miyake M, Togawa N, Ninomiya K and Higashino K (1998) Interleukin 6 and its relationship to clinical parameters in patients with malignant pleural mesothelioma. *Brit J Cancer* **77**: 907–912

O'Byrne K, Dobbs N, Propper D, Smith K and Harris A (1999) Vascular endothelial growth factor, platelet counts, and prognosis in renal cancer. *Lancet* **353**: 1494–1495

O'Byrne KJ, Dalgleish AG, Browning MJ, Steward WP and Harris AL (2000) The relationship between angiogenesis and the immune response in carcinogenesis and the progression of malignant disease. *Euro J Cancer* **36**: 151–169

Ohta Y, Shridhar V, Bright RK, Kalemkerian GP, Du W, Carbone M, Watanabe Y and Pass HI (1999) VEGF and VEGF type C play an important role in angiogenesis and lymphangiogenesis in human malignant mesothelioma tumours. *Brit J Cancer* **81**: 54–61

O'Reilly M, Holmgren L, Shing Y, Chen C, Rosenthal R, Moses M, Lane W, Cao Y, Sage E and Folkman J (1994) Angiostatin: a novel angiogenesis inhibitor that mediates the suppression of metastases by a Lewis lung carcinoma. *Cell* **79**: 315–328

O'Reilly M, Boehm T, Shing Y, Fukai N, Vasios G, Lane W, Flynn E, Birkhead J, Olsen B and Folkman J (1997) Endostatin: an endogenous inhibitor of angiogenesis and tumor growth. *Cell* **88**: 277–285

Peto J, Decarli A, La Vecchia C, Levi F and Negri E (1999) The European mesothelioma epidemic. *Brit J Cancer* **79**: 666–672

Pinedo H, Verheul H, D'Amato R and Folkman J (1998) Involvement of platelets in tumour angiogenesis? *Lancet* **352**: 1775–1777

Procopio A, Strizzi L, Vianale G, Betta P, Puntoni R, Fontana V, Tassi G, Gareri F and Mutti L (2000) Simian virus-40 sequences are a negative prognostic cofactor in patients with malignant pleural mesothelioma. *Genes Chromosomes Cancer* **29**: 173–179

Rusch V (1995) A Proposed New International TNM Staging System for Malignant Pleural Mesothelioma. *Chest* **108**: 1122–1128

Schouwink H, Korse CM, Bonfrer JM, Hart AA and Baas P (1999) Prognostic value of the serum tumour markers Cyfra 21–1 and tissue polypeptide antigen in malignant mesothelioma. *Lung Cancer* **25**: 25–32

Scott HR, McMillan DC, Crilly A, McArdle CS and Milroy R (1996) The relationship between weight loss and interleukin 6 in non-small-cell lung cancer. *Br J Cancer* **73**: 1560–1562

Shetty S, Kumar A, Johnson A, Pueblitz S and Idell S (1995) Urokinase receptor in human malignant mesothelioma cells: role in tumor cell mitogenesis and proteolysis. *Am J Physiology* **268**: L972–L982

Sterman DH, Kaiser LR and Albelda SM (1999) Advances in the treatment of malignant pleural mesothelioma. *Chest* **116**: 504–520

Steward W (1999) Marimastat (BB2516): Current status of development. *Cancer Chemotherapy and Pharmacology* **43 (suppl)**: S56–S60

Sugarbaker DJ, Flores RM, Jaklitsch MT, Richards WG, Strauss GM, Corson JM, DeCamp MM, Jr, Swanson SJ, Bueno R, Lukanich JM, Baldini EH and Mentzer SJ (1999) Resection margins, extrapleural nodal status, and cell type determine postoperative long-term survival in trimodality therapy of malignant pleural mesothelioma: results in 183 patients. *J Thorac Cardiovas Surg* **117**: 54–63; discussion 63–65

Talks KL and Harris AL (2000) Current status of antiangiogenic factors. *Br J Haematol* **109**: 477–489

Tolnay E, Kuhnen C, Wiethege T, Konig JE, Voss B and Muller KM (1998) Hepatocyte growth factor/scatter factor and its receptor c-Met are overexpressed and associated with an increased microvessel density in malignant pleural mesothelioma. *J Cancer Res Clin Oncol* **124**: 291–296

Twardowski P and Gradishar W (1997) Clinical trials of antiangiogenic agents. *Curr Opin Oncol* **9**: 584–589

Verheul HM, Hoekman K, Lupu F, Broxterman HJ, van der Valk P, Kakkar AK and Pinedo HM (2000) Platelet and coagulation activation with vascular endothelial growth factor generation in soft tissue sarcomas. *Clin Cancer Res* **6**: 166–171

Vermeulen P, Gasparini G, Fox S, Toi M, McCulloch P, Pezzella F, Viale G, Weidner N, Harris A and Dirix L (1996) Quantification of Angiogenesis in Solid Human Tumours: an International Consensus on the Methodology and Criteria of Evaluation. *Euro J Cancer* **32A**: 2474–2484

Vermeulen P, Salven P, Benoy I, Gasparini G and Dirix L (1999) Blood platelets and serum VEGF in cancer patients. *Br J Cancer* **79**: 370–376

 *© 2001 Cancer Research Campaign*
